# Understanding the Differentiation, Expansion, Recruitment and Suppressive Activities of Myeloid-Derived Suppressor Cells in Cancers

**DOI:** 10.3390/ijms21103599

**Published:** 2020-05-20

**Authors:** Hui Xuan Lim, Tae Sung Kim, Chit Laa Poh

**Affiliations:** 1 Centre for Virus and Vaccine Research, School of Science and Technology, Sunway University, Bandar Sunway, Kuala Lumpur, Selangor 47500, Malaysia; huixuanl@sunway.edu.my; 2 Division of Life Sciences, College of Life Sciences and Biotechnology, Korea University, Seoul 136-701, Korea; tskim@korea.ac.kr

**Keywords:** MDSC subsets, G-MDSCs, M-MDSCs, cancers, immunosuppression

## Abstract

There has been a great interest in myeloid-derived suppressor cells (MDSCs) due to their biological functions in tumor-mediated immune escape by suppressing antitumor immune responses. These cells arise from altered myelopoiesis in response to the tumor-derived factors. The most recognized function of MDSCs is suppressing anti-tumor immune responses by impairing T cell functions, and these cells are the most important players in cancer dissemination and metastasis. Therefore, understanding the factors and the mechanism of MDSC differentiation, expansion, and recruitment into the tumor microenvironment can lead to its control. However, most of the studies only defined MDSCs with no further characterization of granulocytic and monocytic subsets. In this review, we discuss the mechanisms by which specific MDSC subsets contribute to cancers. A better understanding of MDSC subset development and the specific molecular mechanism is needed to identify treatment targets. The understanding of the specific molecular mechanisms responsible for MDSC accumulation would enable more precise therapeutic targeting of these cells.

## 1. Developmental Origin of Myeloid-Derived Suppressor Cells

Myeloid-derived suppressor cells (MDSCs) represent a population of heterogeneous myeloid lineage cells that have the potent immunosuppressive activity of T cell activation and function. They comprise macrophages, granulocytes, and dendritic cells in immature stages of development. Hematopoietic stem cells give rise to myeloid progenitor and precursor cells in the bone marrow. These immature myeloid cells (IMCs) migrate to peripheral lymphoid organs and differentiate into mature granulocytes, macrophages, or dendritic cells. MDSCs arise from common myeloid progenitors and are arrested in an immature phase of differentiation. Various sources of immunological stress, including cancer, chronic inflammation, trauma, and autoimmune disorder, can inhibit the differentiation and promote the expansion of IMCs. IMCs can be activated by tumor-derived factors and host cytokines, which lead to the generation of MDSCs with potent immunosuppressive potential [1]. Healthy people do not have MDSCs, but in pathological conditions, MDSCs can be detected in the bone marrow, spleen, blood, tumor, and lymph nodes. The frequency of circulating MDSC increases dramatically in cancer, autoimmunity, infection, always correlates with the disease severity and worsens the survival rates.

## 2. MDSC Surface Markers and Subsets

MDSCs are characterized by the co-expression of surface markers GR-1 and CD11b in mice. Normal mouse bone marrow contains 20–30% of cells with this phenotype, but only approximately 2–4% of cells are present in the spleen, and these cells are absent from the lymph nodes. In naive mice, CD11b^+^ GR1^+^ lacks immunosuppressive activity, but these cells have strong immunosuppressive effects on T cell response in tumor-bearing mice. Since GR-1 antibodies can bind to two separate epitopes, Ly6G and Ly6C, these epitope-specific antibodies have been used to distinguish two MDSC subsets: granulocytic MDSCs (G-MDSCs), which have a CD11b^+^Ly6G^+^Ly6C^low^ phenotype, whilst monocytic MDSCs (M-MDSCs) have a CD11b^+^Ly6G^−^Ly6C^high^ phenotype [2]. CD49d was suggested by Haile et al., 2010, to be an alternative marker for Gr-1 to differentiate G-MDSCs (CD11b^+^ CD49d^−^) and M-MDSCs (CD11b^+^ CD49d^+^) [3] (Table 1). Evidence indicates that both MDSC subsets expanded in most of the murine tumor model, but the expansion of G-MDSCs was much greater than the M-MDSCs and represented more than 80% of all MDSCs [4,5]. The frequency of G-MDSCs was also greater than M-MDSCs in the peripheral blood and tumor tissue of pancreatic cancer patients [6]. However, studies have shown that M-MDSCs have higher suppressive activity than G-MDSCs on a single-cell basis [7]. Additionally, M-MDSCs acquired the ability to differentiate into tumor-associated macrophages (TAMs), which produced immunosuppressive cytokines that protected the tumor from the immune system and immunotherapy [8]. Another subset of MDSCs resembles eosinophils, Eo-MDSC, (CD11b^+^Syglec-F^+^CCR3^low^IL-5Ra^low^SSC-A^high^) was identified in mice with chronic *Staphylococcus aureus* infection [5]. Human MDSC was firstly identified in hepatocellular carcinoma and non-Hodgkin’s lymphoma patients with phenotypes CD14^+^HLA-DR^low/−^ [9,10]. Other phenotypic markers for human MDSC subsets in the peripheral blood include CD11b^+^CD14^–^CD15^+^ or CD11b^+^CD14^−^CD66b^+^ for G-MDSC, CD11b^+^CD14^+^HLA-DR^−/low^CD15^−^ for M-MDSC, and Lin^−^HLA-DR^−^CD33^+^ for more immature MDSC progenitors (Table 1) [11]. However, some of the markers mentioned earlier overlapped with other cell populations. Hence, phenotypic characterization in combination with immune-suppressive activity is the optimal strategy for identifying MDSCs.

G-MDSCs and neutrophils are phenotypically and morphologically similar. The main feature of G-MDSCs, which differs from neutrophils, is their suppressive activity. Recently, more approaches were used to distinguish these cells based on genomic, proteomic, and biochemical characteristics. Clinically, an elevated neutrophil/lymphocyte ratio (NLR) has been reported to relate to poor prognosis in several cancers including prostate cancer, gastric cancer, lung cancer, and ovarian cancer patients [13,14,15,16]. G-MDSCs could be considered as pathologically activated neutrophils. Chen et al., 2018, reported that the NLR positively correlated with MDSC levels in the circulation and the prognosis of head and neck squamous cell carcinoma [17]. Other studies have also reported that the MDSC levels correlated with NLR in metastatic prostate cancer and urothelial carcinoma patients [12,18]. However, these authors did not specify which MDSC subset (granulocytic or monocytic myeloid cells) contributed to the overall NLR.

## 3. Factors Affecting MDSC Differentiation and Expansion

**Figure 1:**
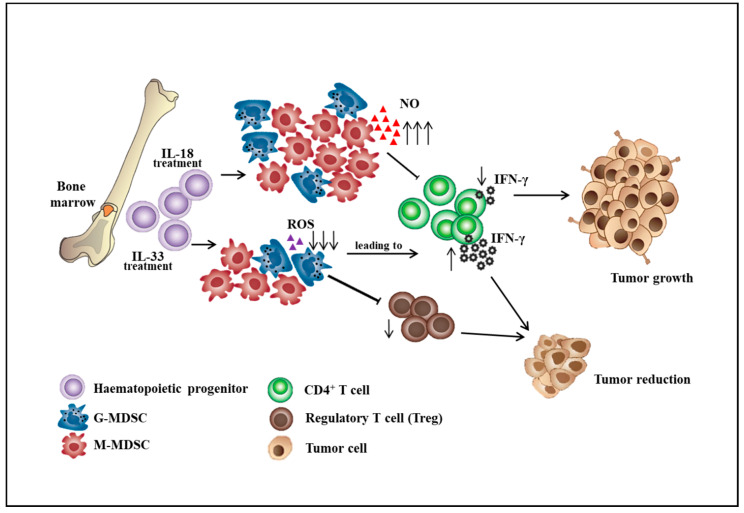
The roles of interleukin-18 and interleukin-33 on the differentiation of bone marrow cells into myeloid-derived suppressor cell subsets. ↑: increase level, ↓: decrease level.

MDSCs participate in immunosuppression by inhibiting the effector function of T cells in the tumor microenvironment, thereby influencing the effectiveness of cancer immunotherapy. The effort to improve the ability of effector T cells to kill tumors will not be sufficient in the immunosuppressive tumor microenvironment consisting of MDSCs, tumor-associated macrophages (TAMs), cancer-associated fibroblasts (CAFs), and T regulatory cells (Tregs). The strategy that alters the differentiation, expansion, and function of MDSCs can partially restore anti-tumor immunity. The differentiation of MDSCs could be driven by various mediators including GM-CSF, G-CSF, M-CSF, VEGF, SCF, IL-6, and IL-13 [19,20]. Immunosuppressive cytokines such as soluble tumor necrosis factor (sTNF), IL-1β, transforming growth factor β (TGF-β), and IL-10 could subvert the immunosurveillance [21,22]. For example, sTNF binding phosphorylated the signal transducer and activator of transcription 3 (STAT3), inducing the proliferation and differentiation of myeloid precursors into MDSCs [23]. TGF-β increased the expansion of the M-MDSC population, the expression of immunosuppressive molecules by MDSCs, and the ability of MDSCs to suppress CD4^+^ T cell proliferation [24]. IL-10 produced by myeloid-derived suppressor cells is critical for the induction of Tregs, which provides a link between different suppressive cells in the tumor microenvironment [25]. Besides, IL-18 was shown to promote the differentiation of CD11b^−^ bone marrow progenitor cells into M-MDSCs. IL-18–induced MDSCs showed enhanced suppression of CD4^+^ T cell proliferation and IFN-γ secretion along with a significant increase of M-MDSC suppressive function, including NO production and arginase 1 expression [26]. However, IL-33 was shown to reduce the differentiation of lineage negative bone marrow precursor cells into G-MDSCs. IL-33 treatment of hematopoietic CD11b^−^ cells sorted from the bone marrow resulted in a marginal decrease in the percentage of G-MDSCs. Importantly, IL-33 treatment significantly impaired the immunosuppressive capacity of MDSCs by reduced inhibition of T cell proliferation and IFN-γ production and also decreased the capacity to induce the differentiation or expansion of Treg cells (Figure 1) [27]. Additionally, aminoacyl-tRNA synthetase-interacting multifunctional protein 1 (AIMP1), a novel pleiotropic cytokine, was shown to inhibit the expansion of MDSCs and tumor growth by reducing the MDSCs in tumor tissues. AIMP1 was suggested to inhibit the immunosuppressive function of M-MDSCs due to the reduction of NO production and arginase activity [28].

Other molecules including prostaglandin E2, S100A8/9 proteins, toll-like receptor agonists, tumor-derived exosome-associated Hsp72, inflammasome component NLRP3, complement component C5a, and vasoactive intestinal peptide have also been shown to contribute to MDSC differentiation [1,29,30,31,32,33,34,35]. For example, tumor-derived factors promoted MDSC differentiation by inducing the intracellular production of PGE2 [36,37]. COX-2 induction is associated with an increased production of PGE2; therefore, COX-2 blockade was shown to suppress tumor by reducing the MDSC-attracting chemokine CCL2 and the number of G-MDSCs in the tumor microenvironment [38]. Most of these soluble factors have been identified to be secreted by a wide range of cancer cell lines in vitro and then distributed through the circulation to bone marrow. Hypoxia-inducible factor (HIF)-1α was found to alter the function of MDSC dramatically in the tumor microenvironment and redirected their differentiation towards tumor-associated macrophages [39,40]. The transcription factor NFIA has been shown to diminish the expression of miR-223 with crucial functions in myeloid lineage development. Conditional deletion of the NFIA gene in the myeloid lineage precludes MDSC development. NFIA-deficient Gr1^+^CD11b^+^ myeloid cells are not immunosuppressive and differentiate normally into macrophages and dendritic cells. NFIA could attenuate monocytic and granulocytic differentiation by downregulating the expression of the M-CSF receptor and the G-CSF receptor on human hematopoietic progenitors [41].

## 4. Factors Affecting MDSC Recruitment

The major role of chemokines is to act as a chemoattractant to guide the migration of cells. MDSCs were demonstrated to be recruited to the tumor site by chemokines CCL2, CXCL5, and CXCL12 [21]. The importance of CXCL-1, CCL5, and CCL7 in MDSC enrichment was also demonstrated in murine colon and liver carcinoma models [33]. PGE2 was reported to promote the accumulation of human MDSCs in the ovarian and gastric cancer microenvironment by enhancing the production of CXCL12 and CXCR4 expression [42]. CCL2-CCR2 signaling has been shown to recruit MDSCs into the tumor microenvironment to suppress antitumor immune responses [43]. It was also reported that CCL5 promoted VEGF-dependent tumor angiogenesis in the human osteosarcoma microenvironment by activating the hypoxia-inducible factor (HIF)-1α signaling cascades [44].

Studies indicated that the types of chemokines responsible for the recruitment of MDSC into the tumor site were dependent on the different MDSC subsets and tumor models. CCL2 signaling has been shown to accumulate M-MDSCs in multiple tumor models [45]. M-MDSCs have been shown to depend on CCR2-mediated signals in regulating the entry of CD8^+^ T cells into the tumor site in melanoma patients [46]. Additionally, Schlecker et al., 2012, reported that tumor-infiltrating M-MDSCs produced high levels of the CCR5 ligands CCL3, CCL4, and CCL5 and recruited high numbers of Tregs into the tumor microenvironment [47]. Other investigators also reported the role of CCL3, CCL5, and CX3CL1 in the migration of M-MDSC [48].

G-MDSCs are recruited primarily by CXC chemokines, which include CXCL1, CXCL2, and CXCL5. Their receptor CXCR2 was specifically expressed in G-MDSC purified from RET.AAD tumor with spontaneous melanoma. RET.AAD mice are transgenic for the human RET oncogene and the chimeric mouse/human MHC antigen AAD. Genetic deletion of CXCR2 impaired the recruitment of G-MDSCs to the primary tumor in vivo [49]. The knockdown of CCL15 in colorectal cancer cells was shown to diminish CCR1^+^ accumulation, and tumor growth was suppressed. Most of the CCR1^+^ cells were G-MDSCs, and the CCL15 levels in the sera of colorectal cancer patients were significantly higher than those in controls [50].

## 5. Suppressive Mechanisms of MDSC

MDSCs have potent immunosuppressive activities and can impair both the innate and adaptive immune responses. They affect the innate immunity by secreting immunosuppressive cytokines such as IL-10 and TGF-β, driving macrophages to exhibit a suppressive M2 phenotype, and negatively regulate the maturation of natural killer cells. MDSCs can inhibit DC maturation by reducing antigen uptake and prevent migration of immature and mature DCs. They can block the ability of DCs to induce IFN-γ-producing T cells and skewing DC cytokine production towards an anti-inflammatory phenotype [51]. MDSCs are known to inhibit adaptive immunity by suppressing T cell activation, proliferation, and function. The depletion of the essential amino acid L-arginine was reported to lead to the loss of the T cell receptor (TCR)z chain, resulting in T cell anergy [49]. Besides, MDSCs were shown to promote the formation of Treg cells and the differentiation of fibroblasts to cancer-associated fibroblasts (CAFs) [52].

**Figure 2:**
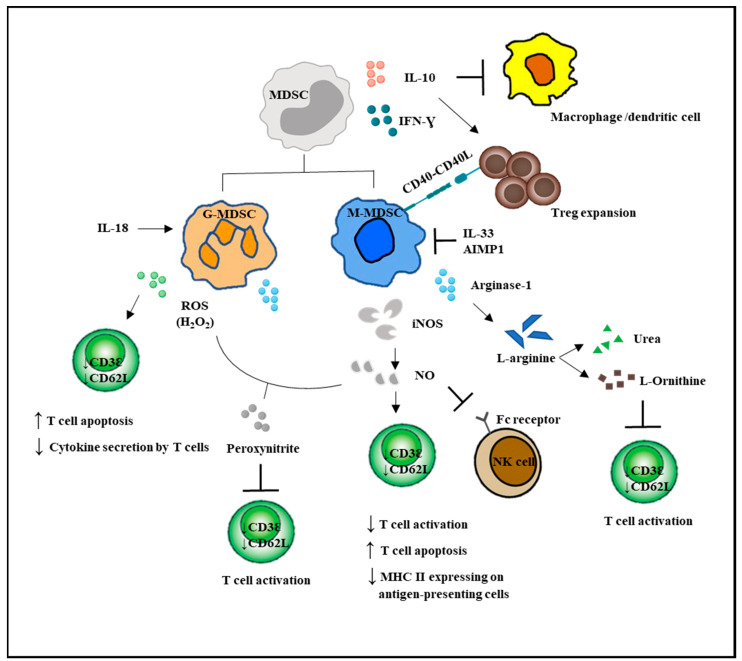
MDSC-mediated immunosuppression in innate and adaptive immune responses. MDSCs suppressed the activation of macrophages and the antigen-presenting ability of the dendritic cells. MDSCs enhanced Treg expansion and suppressed NK cell cytotoxicity. Direct actions of MDSCs on T cells are by increased NO and ROS secretion and decreased L-arginine production. T bar refers to inhibition.

G-MDSCs and M-MDSCs inhibit T cell function via different mechanisms (Figure 2). G-MDSC has increased NADPH oxidase (Nox) activity, which results in high levels of ROS, but low levels of nitric oxide (NO) production. M-MDSCs express high levels of NO, but show low ROS production. Both G-MDSC and M-MDSC subsets express arginase 1. ROS produced by G-MDSCs in high concentrations not only induced T cell apoptosis, but has been demonstrated to cause T cell anergy by downregulating the expression of TCR ζ-chain, leading to impaired TCR signaling [5,11,53]. Besides, ROS form peroxynitrite, which when reacted with NO, nitrosylated the TCR and could result in T cell anergy. NO production by M-MDSCs could induce T cell anergy and nitrosylate important mediators of the IL-2 pathway [54]. Recently, NO production was shown to impair Fc receptor-mediated natural killer cell function, leading to impaired response to monoclonal antibody therapy in cancer [55]. Another important immunosuppressive mediator arginase 1 was able to convert L-arginine into L-ornithine and urea, leading to the depletion of L-arginine. The lack of L-arginine caused a translational blockade in infiltrating T cells and led to cell cycle arrest in G0-G1 [56].

Recently, long noncoding RNA Pvt1 (lncRNA Pvt1) was suggested to be a potent antitumor immunotherapy target. The knockdown of lncRNA Pvt1 significantly reduced the immunosuppressive activity of G-MDSCs such as decreased levels of Arg1 and ROS in G-MDSCs and delayed tumor progression in tumor-bearing mice [57]. Veglia et al., 2019, discovered that fatty acid transport protein 2 (FATP2) controlled the suppressive activity of G-MDSCs via increased uptake of arachidonic acid and the synthesis of PGE2. Overexpression of FATP2 in G-MDSCs was induced by GM-CSF, through the activation of the STAT5 transcription factor. Inhibition of FATP2 abrogated the activity of G-MDSCs and substantially delayed tumor progression [58]. The Olfr29-ps1 pseudogene was reported to be expressed in MDSCs and upregulated by the proinflammatory cytokine IL-6. Olfr29-ps1 promoted the immunosuppressive function and differentiation of M-MDSCs through downregulation of miR-214-3p, thereby releasing the expression of its target gene MyD88 in response to inflammatory factors [59].

Additionally, MDSCs were reported to exert their immunosuppressive effects via the upregulation of programmed death-ligand 1 (PD-L1) [40]. The binding of PD-L1 to the programmed cell death protein 1 (PD-1) receptor expressed on T cells caused exhaustion of T cells, and they lost their ability to produce interferon IFN-γ and IL-2 [60]. Recently, Strauss et al., 2020, discovered the role of PD-1 expressed by myeloid cells in dampening antitumor immunity. Deleting PD-1 from myeloid cells when compared to deleting it from T cells in mice led to a more significant reduction in tumor growth [61]. Moreover, MDSCs were shown to express the death receptor CD95 and induced T cell apoptosis via CD95 ligands expressed on activated T cells [62].

MDSCs are known to affect both the innate and adaptive immune responses [30]. These cells induce anergy of NK cells as an immune evasion mechanism [63]. Macrophages are also downregulated by MDSCs, which results in a decrease in IL-12 production and an increase in IL-10 synthesis [64]. M-MDSCs, but not G-MDSCs, could promote the differentiation of Treg from CD4^+^ T cells. The lack of CD40 on MDSCs resulted in decreasing either the expansion or de novo production of Treg, suggesting that the interaction of co-stimulatory molecules CD40 and CD40L is crucial for Treg development [65]. Additionally, the signal stimulated MDSCs to acquire immunosuppressive properties that were mediated through STAT1, STAT3, STAT6, and NF-κB transcription factors. The mechanisms regulating the activation of M-MDSCs depend on STAT1, STAT6, and NF-κB. STAT3 not only plays a major role in the expansion of both MDSC subsets, but is also involved in the suppressive abilities of G-MDSCs [29].

## 6. MDSC in Cancer Progression

Several lines of evidence indicated that MDSCs were associated with tumor progression. The levels of MDSCs were profoundly correlated with the extent of tumor burden and the overall survival of the tumor-bearing host. Administration of MDSCs in the murine tumor models was found to significantly promote tumor growth [66,67,68]. The detrimental effects of MDSCs in tumor progression have been well described as the depletion of Gr-1^+^ cells in tumor-bearing mice strikingly inhibited tumor growth, reduced cancer cell dissemination a d metastasis, and prolonged survival [63,69]. The reduction of murine MDSC numbers was shown to facilitate the rejection of established metastatic disease after the removal of primary tumors [70]. A study in renal cell carcinoma showed that surgical resection of primary cancer lesion contributed to the reduction of MDSC, which indicated the unknown factors derived from cancer tissue affected MDSC maintenance [71]. Kawano et al., 2015, reported a statistically significant higher frequency of circulating MDSCs in the blood of advanced and recurrent patients with cervical cancers compared to healthy patients. Therefore, circulating MDSCs have been validated as a predictive marker for cancer immunotherapy. Further characterization of MDSC subsets demonstrated that the frequency of both M-MDSCs and G-MDSCs was significantly elevated in the blood of patients from advanced glioma and cervical cancer [12,72]. Recently, a study suggested that G-MDSCs could serve as a potential biomarker for disease progression of cervical cancers. The frequency of circulating G-MDSCs was found to correlate with disease prognosis, while the percentage of M-MDSCs was only elevated in patients with advanced cervical cancers. For patients with early and locally advanced cervical cancers, the frequency of circulating G-MDSCs, but not M-MDSCs correlated with tumor recurrence. The levels of circulating G-MDSCs also negatively correlated with the densities of CD8^+^ T cells and the suppression of T cell proliferation [12]. M-MDSCs were reported as prognosis markers in the, colorectal, gastric, and pancreatic cancer [73,74]. Increased levels of M-MDSCs in advanced non-small cell lung cancer (NSCLC) patients were associated with an unfavorable clinical outcome [75]. The lower quantity of M-MDSCs in metastatic melanoma patients following treatment with ipilimumab were more likely to achieve prolonged survival [76]. Therefore, MDSC could serve as a predictive marker for immunotherapy.

MDSCs and their immunosuppressive functions might be eliminated via several approaches such as deactivation of MDSCs, promoting the differentiation of MDSCs into mature cells, blocking the development of MDSCs, and depletion of MDSCs [77]. MDSCs could be inactivated by blocking the NO, ROS, and arginase secretion such as by using phosphodiesterase inhibitors, nitroaspirins, synthetic triterpenoids, COX2 inhibitors, ARG1 inhibitors, anti-glycan antibodies, CSF-1R, IL-17, and histamine inhibitors. Agents that block the development of MDSCs include N-bisphosphonates, modulators of tyrosine kinases, and STAT3 inhibitors. MDSCs could also be depleted with gemcitabine, HSP90 (heat shock protein 90) inhibitors, and paclitaxel (Table 2). Some FDA-approved compounds such as ATRA (All-trans retinoic acid), PDE5 (phosphodiesterase type 5) inhibitors, COX-2 inhibitors, or bisphosphonates are already in clinical trials for evaluating their ability to inhibit MDSCs and enhance anti-tumor immunity in humans (Table 2) [78]. However, major anti-tumor effects may not be expected by only targeting MDSCs. It has been found that gemcitabine and anti-GR-1 Ab, when administered together with DNA vaccine, could induce a strong antitumor immune response, which was accompanied by reduced self-tolerance in a preclinical HER2-expressing mouse tumor model [79]. ATRA is a promising agent that promoted the differentiation of M-MDSCs into mature cells, and when used together with a dendritic cell (DC) vaccine against p53, substantial improvement of the CD8^+^ T cell responses was observed in late-stage small cell lung cancer patients [80].

The combination of MDSC targeting with immune checkpoint inhibitors has been applied in preclinical tumor models and cancer patients. The FDA approved immune checkpoint inhibitors including one CTLA-4 inhibitor (ipilimumab), three PD-1 inhibitors (nivolumab, pembrolizumab, and cemiplimab), and three PD-L1 inhibitors (atezolizumab, durvalumab, and avelumab). Serine/threonine protein kinase CK2 inhibition blocked MDSC differentiation and substantially increased anti-tumor efficacy when combined with anti-CTLA-4 blockade in mice [81]. Treatment of tumor-bearing mice with Sema4D mAb in combination with either CTLA-4 or PD-1 blockade enhanced the rejection of tumors or tumor growth delay, resulting in prolonged survival with either treatment [82]. A recent study has shown that the combination of anti-CXCR4, which decreased M-MDSC, and anti-PD-1 therapy improved the overall survival in a mouse glioma model [83]. Furthermore, ATRA decreased the frequency of circulating MDSCs in melanoma patients treated in combination with ipilimumab, and this combination is still on-going in a clinical trial to treat Stage IV melanoma patients [84,85].

## 7. Conclusions

Successful immunotherapeutic approaches that utilize the host immune system to inhibit tumor growth could lead to increased patient survival. These approaches are non-toxic and usually involve either regulation of the secretion of soluble factors such as cytokines, chemokines, and tumor-derived factors by immune cells or a reduction in the activity of immune regulatory cells such as regulatory T (Treg) cells and myeloid-derived suppressor cells (MDSCs). The depletion of MDSCs normally involved cytotoxic agents, but an alternative approach might be better by altering the development, differentiation, and functions of MDSCs. PD-1 expression from myeloid cells plays a critical role in preventing the differentiation of effector myeloid cells and promoting the formation of MDSCs; thus, blocking PD-1 signaling in myeloid cells appears to be a requirement for antitumor immunity. Additionally, combinatorial approaches to target precisely the suppressive activity of MDSCs or MDSC subsets using anti-IL-18, inhibition of FATP2, inactivating the long non-coding RNA Pvt1, the NFIA gene, or the Olfr19-ps1 pseudogene might enhance the existing immunotherapeutic strategies by the administration of immune checkpoint inhibitors including the CTLA-4, PD-1, and PD-L1 inhibitors.

## Figures and Tables

**Table 1:** Phenotype and functional proteins of murine and human MDSCs.

MDSC Subsets		Phenotype	References
**Murine**	MDSC G-MDSC M-MDSC	CD11b^+^ GR1^+^ CD11b^+^ Ly6G^+^Ly6C^low^ CD11b^+^ Ly6G^neg^Ly6C^high^	[2]
**Murine**	G-MDSC M-MDSC	CD11b^+^ CD49^−^ CD11b^+^ CD49^+^	[3]
**Human**	MDSC G-MDSC M-MDSC	CD14^+^HLA-DR^low/−^ CD14^−^CD11b^+^CD33^+^CD15^+^ CD11b^+^ HLA-DR^low/−^CD14^+^	[10]
**Human**	G-MDSC M-MDSC	CD11b^+^CD14^–^CD15^+^ CD11b^+^CD14^–^CD66b^+^ CD11b^+^CD14^+^HLA-DR^−/low^CD15^−^	[11]
**Human**	MDSC G-MDSC M-MDSC	Lin^−^HLA-DR^−^CD11b^+^CD33^+^ HLA-DR^−^CD11b^+^CD14^−^CD15^+^CD33^+^ HLA-DR^−^CD11b^+^CD14^+^CD15^−^CD33^+^	[12]

**Table 2:** Strategies for myeloid-derived suppressor cell (MDSC) targeting.

Strategy	Mechanism of Action	Examples	Clinical Trial
Blocking MDSC development	N-Bisphosphonates	Zoledronic acid	Phase 3-completed
	Multi-kinase inhibitors	Sunitinib Sorafenib	Phase 2-completed Phase 3-completed
	JAK2/STAT3 inhibitors	Cucurbitacin B JSI-124	N/A N/A
	Blocking antibodies	Anti-VEGF antibodies	NCT03503604
Differentiation of MDSC into mature cells	Vitamins	ATRA Vitamin A Vitamin D3 Vitamin E	NCT024403778 N/A N/A N/A
	Cytokines	IL-12	N/A
	Others	CpG	N/A
MDSC deactivation	PDE5 inhibitors	Sildenafil Tadalafil	NCT02544880 NCT01697800
	NO inhibitors	NO-aspirins (NCX-4016) L-NAME	Phase1-completed
	ROS inhibitors	Synthetic triterpenoids (omaveloxolone)	Phase 2-completed
	Arginase inhibitors	COX2 inhibitors NOHA L-NAME	N/A N/A N/A
	Recruitment and migration inhibitor	Anti-glycan antibodies CSF-1R inhibitors	NCT03557970
	Others	Histamine inhibitor (ranitidine) Anti-IL-17 antibodies	NCT03145012
MDSC depletion	Cytotoxic agents	Gemcitabine Cisplatin 5-Fluorouracil Paclitaxel	NCT01803152 NCT02432378 N/A N/A
	HSP90 inhibitors	17-DMAG	Phase 1-completed
	Peptide-FC fusion proteins	N/A	N/A

N/A refers to no available information. ATRA: All-trans retinoic acid; PDE5: phosphodiesterase type 5; NCX: Nitric Oxide-Aspirin; L-NAME: L-N^G^-Nitroarginine methyl ester; NOHA: N(omega)-hydroxy-l-arginine; HSP90: heat shock protein 90; 17-DMAG: 17-Dimethylaminoethylamino-17-demethoxygeldanamycin.
